# Uncertainty-aware deep learning in healthcare: A scoping review

**DOI:** 10.1371/journal.pdig.0000085

**Published:** 2022-08-10

**Authors:** Tyler J. Loftus, Benjamin Shickel, Matthew M. Ruppert, Jeremy A. Balch, Tezcan Ozrazgat-Baslanti, Patrick J. Tighe, Philip A. Efron, William R. Hogan, Parisa Rashidi, Gilbert R. Upchurch, Azra Bihorac

**Affiliations:** 1 Department of Surgery, University of Florida Health, Gainesville, Florida, United States of America; 2 Intelligent Critical Care Center, University of Florida, Gainesville, Florida, United States of America; 3 Department of Biomedical Engineering, University of Florida, Gainesville, Florida, United States of America; 4 Department of Medicine, University of Florida Health, Gainesville, Florida, United States of America; 5 Departments of Anesthesiology, Orthopedics, and Information Systems/Operations Management, University of Florida Health, Gainesville, Florida, United States of America; 6 Department of Health Outcomes & Biomedical Informatics, College of Medicine, University of Florida, Gainesville, Florida, United States of America; 7 Departments of Biomedical Engineering, Computer and Information Science and Engineering, and Electrical and Computer Engineering, University of Florida, Gainesville, Florida, United States of America

## Abstract

Mistrust is a major barrier to implementing deep learning in healthcare settings. Entrustment could be earned by conveying model certainty, or the probability that a given model output is accurate, but the use of uncertainty estimation for deep learning entrustment is largely unexplored, and there is no consensus regarding optimal methods for quantifying uncertainty. Our purpose is to critically evaluate methods for quantifying uncertainty in deep learning for healthcare applications and propose a conceptual framework for specifying certainty of deep learning predictions. We searched Embase, MEDLINE, and PubMed databases for articles relevant to study objectives, complying with PRISMA guidelines, rated study quality using validated tools, and extracted data according to modified CHARMS criteria. Among 30 included studies, 24 described medical imaging applications. All imaging model architectures used convolutional neural networks or a variation thereof. The predominant method for quantifying uncertainty was Monte Carlo dropout, producing predictions from multiple networks for which different neurons have dropped out and measuring variance across the distribution of resulting predictions. Conformal prediction offered similar strong performance in estimating uncertainty, along with ease of interpretation and application not only to deep learning but also to other machine learning approaches. Among the six articles describing non-imaging applications, model architectures and uncertainty estimation methods were heterogeneous, but predictive performance was generally strong, and uncertainty estimation was effective in comparing modeling methods. Overall, the use of model learning curves to quantify epistemic uncertainty (attributable to model parameters) was sparse. Heterogeneity in reporting methods precluded the performance of a meta-analysis. Uncertainty estimation methods have the potential to identify rare but important misclassifications made by deep learning models and compare modeling methods, which could build patient and clinician trust in deep learning applications in healthcare. Efficient maturation of this field will require standardized guidelines for reporting performance and uncertainty metrics.

## Author summary

Deep learning prediction models perform better than traditional prediction models for several healthcare applications. For deep learning to achieve it’s greatest impact on healthcare delivery, patients and providers must trust deep learning models and their outputs. This article describes the potential for deep learning to earn trust by conveying model certainty–the probability that a given model output is accurate. If a model could convey not only it’s prediction but also it’s level of certainty that the prediction is correct, patients and providers could make an informed decision to incorporate or ignore the prediction. The use of uncertainty estimation for deep learning entrustment is largely unexplored, and there is no consensus regarding optimal methods for quantifying uncertainty. Our purpose is to critically evaluate methods for quantifying uncertainty in deep learning for healthcare applications and propose a conceptual framework for specifying certainty of deep learning predictions. We systematically reviewed published scientific literature and summarized results from 30 studies, and found that uncertainty estimation methods have the potential to identify rare but important misclassifications made by deep learning models and compare modeling methods, which could build patient and clinician trust in deep learning applications in healthcare.

## Introduction

Deep learning is increasingly important in healthcare. Deep learning prediction models that leverage electronic health record data have outperformed other statistical and regression-based methods [1,2]. Computer vision models have matched or outperformed physicians for several common and essential clinical tasks, albeit in select circumstances [3,4]. These results suggest a potential role for clinical implementation of deep learning applications in health care.

Mistrust is a major barrier to clinical implementation of deep learning predictions [5,6]. Efforts to restore and build trust in machine learning have focused primarily on improving model explainability and interpretability. These techniques build clinicians’ trust, especially when model outputs and important features correlate with logic, scientific evidence, and domain knowledge [7,8]. Another critically important step in building trust in deep learning is to convey model uncertainty, or the probability that a given model output is inaccurate [8]. Deep learning models that typically perform well make rare but egregious errors [9]. If a model could calculate the uncertainty in its predictions on a case-by-case basis, patients and clinicians would be afforded opportunities to make safe, effective, data-driven decisions regarding the utility of model outputs, and either ignore predictions with high uncertainty or triage them for detailed, human review. Unfortunately, there is a paucity of literature describing effective mechanisms for calculating model uncertainty for healthcare applications, and no consensus regarding best methods exists.

Our purpose is to critically evaluate methods for quantifying uncertainty in deep learning for healthcare applications and propose a conceptual framework for optimizing certainty in deep learning predictions. Herein, we perform a scoping review of salient literature, critically evaluate methods for quantifying uncertainty in deep learning, and use insights gained from the review process to develop a conceptual framework.

## Materials and methods

**Fig 1.**
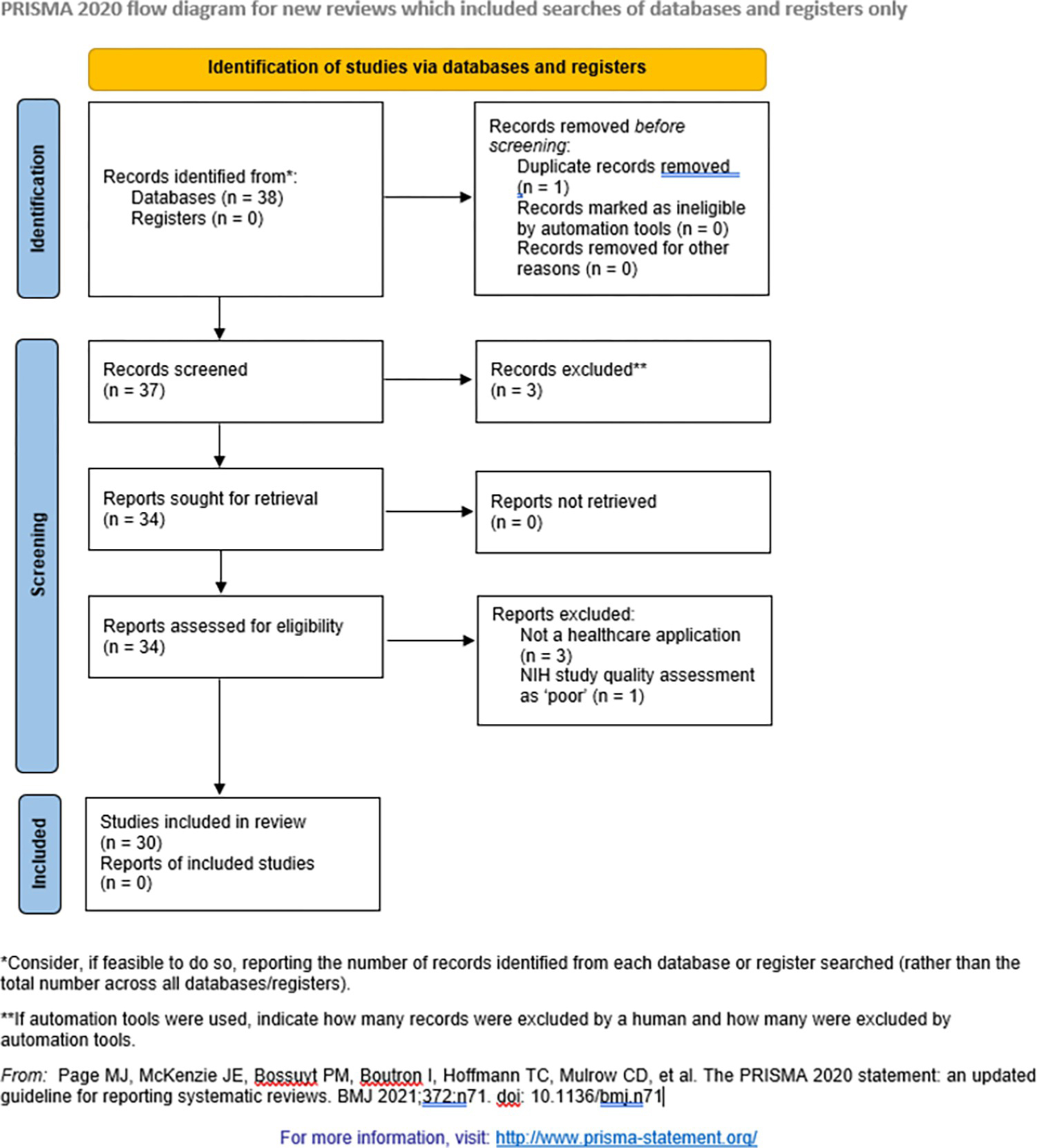
PRISMA flow diagram for article inclusion.

Article inclusion is illustrated in Fig 1, a PRISMA flow diagram. We searched Embase, MEDLINE, and PubMed databases, chosen for their specificity to the healthcare domain, for articles with “deep learning” and “confidence” or “uncertainty” in the title or abstract and for articles with “deep learning” and “conformal prediction” in the title or abstract, identifying 37 unique articles. Two investigators independently screened all article abstracts for relevance to review objectives, removing three articles. Full texts of the remaining 34 articles were reviewed. Study quality was independently rated by two investigators using quality assessment tools specific to the design of the study in question (available at: https://www.nhlbi.nih.gov/health-topics/study-quality-assessment-tools). Only studies describing healthcare applications that were good or fair quality were included in the final analysis, which removed four articles, leaving 30 total articles in the final analysis. Data extraction was performed according to a modification of CHARMS criteria, which included methods for measuring uncertainty in deep learning predictions [10]. The search was performed according to Preferred Reporting Items for Systematic Reviews and Meta-Analyses extension for Scoping Reviews (PRISMA-ScR) guidelines, as listed in S1 PRISMA Checklist.

During screening, there were disagreements between the two investigators regarding the exclusion of five articles; all disagreements were resolved by discussion of review objectives without a third-party arbiter. Cohen’s kappa statistic summarizing interrater agreement regarding article screening was 0.358 (observed agreement = 0.848, expected agreement = 0.764), suggesting that screening agreement between reviewers was fair [11,12]. During full text review, there was a disagreement between the two investigators regarding the exclusion of one article, which was resolved by discussion of review objectives without a third-party arbiter. Cohen’s kappa statistic summarizing interrater agreement regarding full text review could not be calculated because both observed and expected agreement were 0.964, but this high value suggests that agreement between reviewers was substantial.

## Results

Included articles are summarized in Table 1. Notably, the use of uncertainty estimation in these articles was rarely applied to building trust in deep learning among patients, caregivers, and clinicians. Therefore, the presentation of results will focus primarily on the content of the articles, and opportunities to use uncertainty-aware deep learning to build trust will be discussed further in the Discussion section as a novel application of established techniques.

Among 30 included studies, 24 described medical imaging applications and six described non-imaging applications; these categories are evaluated and reported separately. First, important themes from included articles are synthesized into a conceptual framework.

### Conceptual framework for optimizing certainty in deep learning predictions

**Fig 2.**
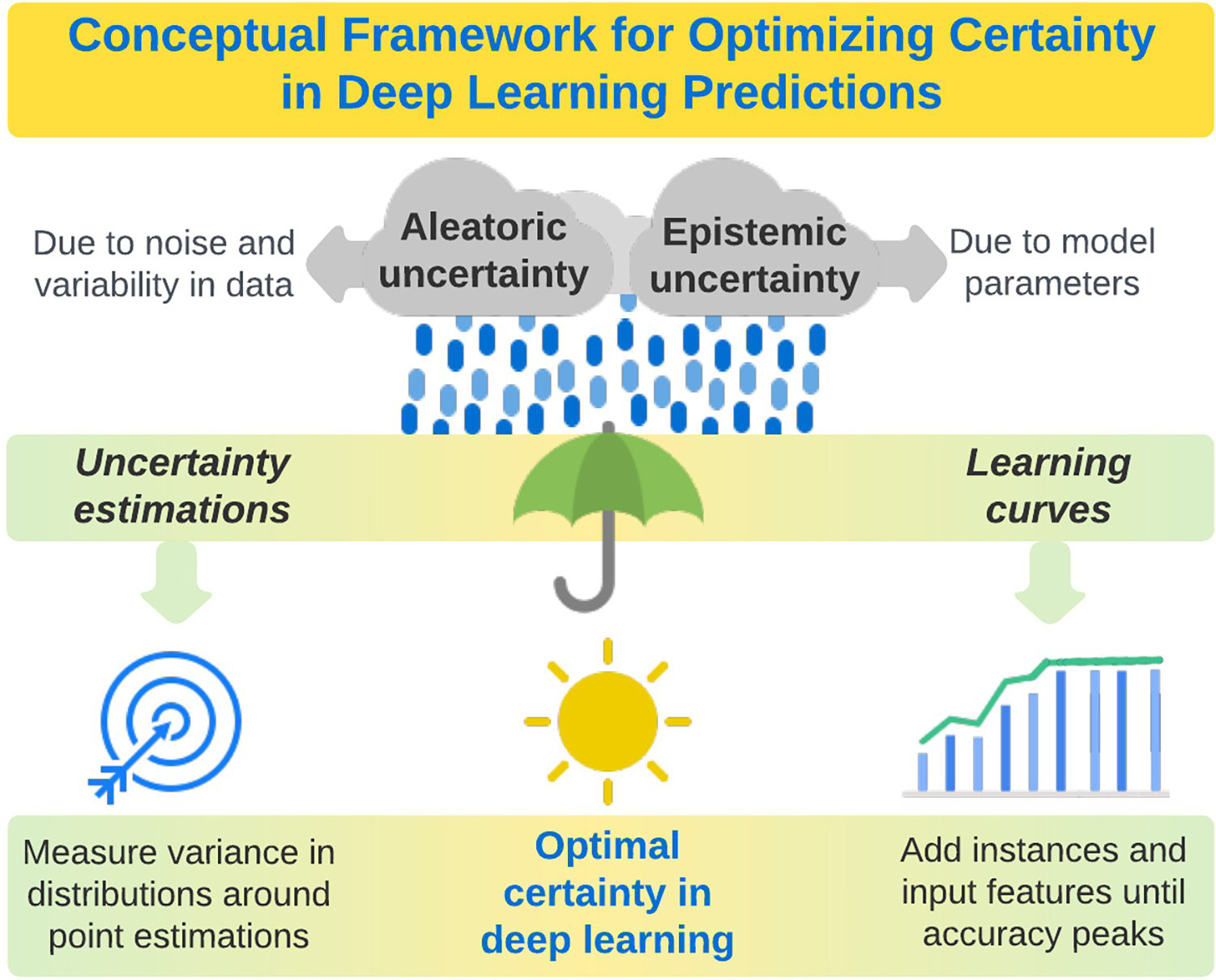
A conceptual framework for optimizing certainty in deep learning predictions by quantifying and minimizing aleatoric and epistemic uncertainty.

Deep learning uncertainty can be classified as epistemic, (i.e., attributable to uncertainty regarding model parameters or lack of knowledge), or aleatoric (i.e., attributable to stochastic variability and noise in data). Epistemic and aleatoric uncertainty have overlapping etiologies, as variability and noise in data can contribute to uncertainty regarding optimal model parameters and knowledge regarding ground truth. In addition, epistemic and aleatoric uncertainty may be amenable to similar mitigation strategies, as collecting and analyzing more data may allow for more effective identification and imputation of outlier and missing values, reducing aleatoric uncertainty, and may also allow for more effective parameter searches. Beyond these overlapping etiologies and mitigation strategies, epistemic and aleatoric uncertainty have some unique and potentially important attributes. Epistemic uncertainty can be seen as a lack of information about the best model and can be reduced by adding more training data [13]. Learning curves stratified by number of training samples offer an intuitive approach to visualizing epistemic uncertainty, where it becomes evident that using more data typically results not only in more accurate models, but also in more stable loss when trained for the same number of epochs. In stochastic models, parameter estimates also become more stable with increasing amounts of training data. In addition to increasing knowledge through larger sample sizes, it may also be possible to reduce epistemic uncertainty by adding input features, especially multi-modal features (e.g., using not only vital signs to predict hospital mortality, but also using laboratory values, imaging data, and unstructured text data from notes written by clinicians), or modifying the algorithm to learn from additional nonlinear combinations of variables. Once an epistemic uncertainty limit has been reached, quantifying the remaining aleatoric uncertainty in predictions could augment clinical application by allowing patients and providers to understand whether predictions have suitable accuracy and certainty for incorporation in shared decision-making, or are too severely compromised by aleatoric uncertainty to be useful, regardless of overall model accuracy [13]. These concepts are illustrated in Fig 2. This explanation considers transforming a given model into a stochastic ensemble through Bernoulli sampling of weights at model test time, giving rise to a measure of epistemic uncertainty for each sample.

### Medical imaging applications

Among the 24 studies describing medical imaging applications, 12 of those 24 (50%) used magnetic resonance imaging (MRI) features for model training and testing; 11 of those 12 (92%) of which involved the brain or central nervous system. The next most common sources of model features were retinal or fundus images (5 of 24, 21%) and endoscopic images of colorectal polyps (3 of 24, 13%). The remaining studies used computed tomography images, breast ultrasound images, lung microscopy images, or facial expressions. All model architectures included convolutional neural networks or a variation thereof (e.g., U-Net).

The predominant method for quantifying uncertainty in model predictions was Monte Carlo dropout, as originally described by Gal and Ghahramani as a Bayesian approximation of probabilistic Gaussian processes [14]. Briefly, during testing, multiple predictions are generated from a given network for which different neurons have dropped out. The neuron dropout rate is calibrated during model development according to training data sparsity and model complexity. Each forward pass uses a different set of neurons, so the outcome is an ensemble of different network architectures that can generate a posterior distribution for which high variance suggests high uncertainty and low variance suggests low uncertainty. Studies assessing the efficacy of uncertainty measurements provided reasonable evidence that uncertainty estimations were useful. In applying a Bayesian convolutional neural network to diagnose ischemic stroke using brain MRI images, Herzog et al [15] found that uncertainty measurements improved model accuracy by approximately 2%. In applying a convolutional neural network to estimate brain and cerebrospinal fluid intracellular volume, Qin et al [16] reported highly significant correlations (all *p*<0.001) between uncertainty estimations and observed error based on ground truth values. Finally, in applying a convolutional neural network for differentiating among glioma, multiple sclerosis, and healthy brain, Tanno et al [17] found that uncertainty-based classification correctly identified 96% of all predictions that had high-risk for error; this error was likely attributable to aleatoric uncertainty from noise and variability in data. Valiuddi et al [18] used Monte Carlo simulations in depicting the performance of a probabilistic U-Net performing density modeling of thoracic computed tomography and endoscopic polyp images, learning aleatoric uncertainty as a distribution of possible annotations using a probabilistic segmentation model. This approach was effective in increasing predictive performance, measured by generalized energy distance and intersection over union, by up to 14%. Collectively, these findings suggest Monte Carlo dropout methods can accurately estimate uncertainty in predictions made by convolutional neural networks that make rare but potentially important misclassifications on medical imaging data, and corroborates prior evidence that Monte Carlo dropout can also offer predictive performance advantages, especially on external validation, by mitigating risk for overfitting.

Conformal prediction–used in two studies–demonstrated strong performance in estimating uncertainty. Wieslander et al [19] applied convolutional neural networks to investigate drug distribution on microscopy images of rat lungs following different doses and routes of medication administration, finding that conformal prediction explained 99% of the variance in predicted versus actual error. In another study by Athanasiadis et al [20], conformal prediction improved audio-visual emotion classification for a semi-supervised generative adversarial network compared with a similar network using the classifier alone.

Two studies used uncertainty estimation to compare modeling methods. Graham et al [21] used uncertainty measurements to demonstrate that a hierarchical approach to labeling regions and sub-regions of the brain produced similar predictive performance with greater certainty compared with a flat labeling approach, at any level of the labeling tree. Alternatively, to evaluate similarity between functional brain networks, Ktena et al [22] use convolutional neural network architectures in deriving a novel similarity metric on irregular graphs, demonstrating improver overall classification. Sedghi et al [23] calculated variance in displacement for different image classifications of brain MRIs, demonstrating good dice values for intra-subject pairs with consistent good results when simulating resections on the images, suggesting utility for challenging clinical scenarios.

### Non-imaging applications

The six studies describing non-imaging medical applications were heterogenous. Five of the studies endeavored to predict and classify biochemical and molecular properties for pharmacologic applications, each with somewhat different model architectures (i.e., ensembles of deep neural networks, convolutional neural networks, and multi-layer perceptrons). Three of these five studies generated posterior distributions and assessed variance across those distributions to approximate prediction uncertainty. In one instance, there was almost no gain in predictive performance; in another by Cortes-Ciriano and Bender, there was strong correlation between estimated confidence levels and the percentage of confidence intervals that encompassed the ground truth (R^2^ > 0.99, *p*<0.001) [24]. This difference in performance may have been attributable to differences in model features. The less successful model used bit strings to represent molecular structures; the more successful model used high-granularity bioactivity features, with 203–5,207 data points per protein. A third study in the molecular property class also used Monte Carlo dropout techniques and reported relatively low test error values [25]. Two studies used conformal predictions to estimate uncertainty, one of which used conformal predictions in predicting active and inactive compound classes, generating single-label predictions for about 90% of all instances with overall confidence 80% or greater. Best results were demonstrated for deep neural networks rather than random forest or light gradient boosting machine models, and conformal prediction offered a controllable error rate and better recall for all three model types [26]. Cortes-Ciriano and Bender [27] leveraged conformal predictions in analyzing errors on ensembles of predictions generated by dropout, reporting strong correlation between confidence levels and error rates (R^2^ > 0.99, p<0.001), with results similar to those reported in their Deep Confidence work [24]. The remaining non-imaging study predicted neurodegenerative disease progression using multi-source clinical, imaging, genetic, and biochemical data, reporting variable predictive performance across different outcomes, but overall strong performance [28]. Compared with the biochemical prediction models, this study used a unique method for quantifying uncertainty, by measuring variance across predictions made by an ensemble of possible patient forecasts using a generative network. Collectively, these findings suggest that unique model architectures and methods for estimating uncertainty can be applied to a variety of non-pixel-based input features, producing occasional predictive performance advantages and accurate uncertainty estimations.

## Discussion

This review found that the uncertainty inherent in deep learning predictions are most commonly estimated for medical imaging applications using Monte Carlo dropout methods on convolutional neural networks. In addition, unique model architectures and uncertainty estimation methods can apply to non-pixel features, simultaneously improving predictive performance (presumably by mitigating risk for overfitting, in the case of Monte Carlo Dropout) while accurately estimating uncertainty. Unsurprisingly, for medical imaging applications, larger datasets of training images were associated with greater predictive performance [15,21,29–38]. We could not perform meta-analyses on predictive performance or uncertainty estimations because performance metrics and methods for quantifying uncertainty were heterogenous, despite relative homogeneity in model architectures–which were primarily based on convolutional neural networks–and homogeneity in methods for estimating uncertainty–which were primarily based on Monte Carlo dropout [14]. Uncertainty estimations for non-medical imaging applications were both sparse and heterogenous. Yet the weight of evidence suggests that a variety of methods can estimate uncertainty in predictions on non-pixel features, offering greater performance and reasonably accurate uncertainty estimations. Conformal prediction demonstrated efficacy in uncertainty estimation as well and is easy to interpret (e.g., at a confidence level of 80%, at least 80% of the predicted confidence intervals contain the true value), and applies not only to deep learning but also to other machine learning approaches such as random forest modeling.

For both imaging and non-imaging applications, uncertainty estimations are poised to augment clinical application by identifying rare but potentially important misclassifications made by deep learning models. First, mistrust of machine learning predictions must be overcome. Model explainability, interpretability, and consistency with logic, scientific evidence, and domain knowledge are critically important in building trust [7,8]. Yet, even when a model is easy to understand, generates predictions consistent with medical knowledge, and has 90% overall accuracy, patients and providers may wonder: is this prediction among the 1 in 10 that is incorrect? Can the model tell me whether it is certain or uncertain of this particular prediction? To address these questions and build trust, it seems prudent to include model uncertainty estimations in shared decision-making processes. Therefore, we believe that uncertainty estimations are a critical element in the safe, effective clinical implementation of deep learning in healthcare. In performing this review, we sought to summarize evidence regarding the efficacy of uncertainty estimation in building trust in deep learning among patients, caregivers, and clinicians, but we found little evidence thereof. Therefore, we propose uncertainty-aware deep learning as a novel approach to building trust.

We found no previous systematic or scoping reviews on the same topic, though several authors have described important components of estimating uncertainty in deep learning predictions. Common statistical measures of spread (e.g., standard deviation and interquartile range) are undefined for single point predictions. Entropy, however, does apply to probability distributions. Therefore, most uncertainty estimation methods generate probability distributions around point estimations. Monte Carlo dropout, as originally described by Gal and Ghahramani, offers an elegant solution [14]. During testing, multiple stochastic predictions are generated from a given network for which different neurons have dropped out with specified probability. This dropout rate is calibrated during model development according to training data sparsity and model complexity. When training, dropping out different sets of neurons at different steps harbors the additional advantage of mitigating overfitting. When testing, each forward pass uses a different set of neurons; therefore, the outcome is an ensemble of different network architectures that can be represented as a posterior distribution. Variance across the distribution of predictions can be analyzed by several methods (e.g., entropy, variation ratios, standard deviation, mutual information). High variance suggests high uncertainty; low variance suggests low uncertainty.

This review was limited by heterogeneity in model performance metrics and methods for quantifying uncertainty. To identify the optimal methods for estimating uncertainty in deep learning predictions, it would be necessary to perform a meta-analysis or comparative effectiveness analyses. This would be facilitated by achieving consensus regarding core performance and uncertainty metrics. The field of deep learning uncertainty estimation is maturing rapidly; it would be advantageous to establish reporting guidelines, as has been done for prediction modeling, causal inference, and machine learning trials [39–42]. Finally, beyond uncertainty estimations, it may be useful to quantify how similar an individual patient is to other patients in the training data, so that users can understand whether uncertainty is attributable to variability in outcomes relative to similar features in the training data or due to a patient having outlier features that are not well represented in the training data.

## Conclusions

For convolutional neural network predictions on medical images, Monte Carlo dropout methods accurately estimate uncertainty. For non-medical imaging applications, a paucity of evidence suggests that several uncertainty estimation methods can improve predictive performance and accurately estimate uncertainty. Using uncertainty estimations to gain the trust of patients and clinicians is a novel concept that warrants empirical investigation. The rapid maturation of deep learning uncertainty estimations in medical literature could be facilitated by achieving consensus regarding performance and uncertainty metrics and standardizing reporting guidelines. Once standardized and validated, uncertainty estimates have the potential to identify rare but important misclassifications made by deep learning models in clinical settings, augmenting shared decision-making processes toward improved healthcare delivery.

## Supplementary Material

Prismap checklist

## Figures and Tables

**Table 1.** Summary of included studies, classified as imaging or non-imaging applications.

Primary author	Purpose	Population or sampling unit	Sample size	Model architecture	Best model performance	Validation method	Method for quantifying prediction uncertainty	Quality Rating
*Medical imaging applications*								
Araújo (34)	Grade diabetic retinopathy severity	Datasets of retinal images	Approximately 93,000 images	Convolutional-batch normalization blocks, max-pooling layers	Quadratic-weighted Cohen’s kappa 0.71–0.84 for predictions vs. ground truth	External	Calculate Cohen’s kappa statistics for model predictions at threshold levels of uncertainty, calculated by variance in image-wise retinopathy grade probability	Good
Athanasiadis (20)	Correlate visual and audio emotional expression	Audio-visual emotion datasets	187 people, 7356 audio recordings, 7442 videos, 96 images	Generative Adversarial Networks	Classification 52.52% in one dataset and 47.11% in the other	External	Conformal prediction to obtain error calibration	Good
Ayhan (31)	Diagnosing diabetic retinopathy	Fundus images	89,215 images	Convolutional neural network	AUC 0.959–0.982	External	Calculate variance in the form of entropy as a distribution of predicted probabilities	Good
Cao (32)	Classify breast masses, identify tumors	Breast ultrasound images	107 patients with 13,382 ultrasound slices	Dense U-Net	Accuracy 99.21%	Internal	Generate visual epistemic uncertainty maps for each image	Fair
Carneiro (29)	Classifying colorectal polyps	Images of colorectal polyps obtained by colonoscopy	940 images from 287 patients	Residual and densely connected convolutional networks	Accuracy 0.76	External	Classification entropy or the predicted variance produced by Bayesian methods	Fair
Edupuganti (35)	Quantify uncertainty in deep MRI segmentation	Knee MRI images	19 patients with 320 2D image slices per patient	Variational autoencoders, convolutional neural networks	R^2^ = 0.97 for 2-fold under sampling	External	Generate a posterior of the MRI image and generate pixel variance maps using Monte-Carlo sampling	Good
Graham (21)	Label regions and sub-regions of the brain	Brain MRI images	593 scans	3D UNet	Dice score 0.845 for all regions in uncertainty-aware hierarchical model	External	Cross-entropy uncertainty measured at each progressively smaller sub-region of the brain	Good
Herzog (15)	Diagnose ischemic stroke	Brain MRI images	511 patients with average 30 images per patient	Bayesian convolutional neural network	Accuracy 95.9%, was 2% better than model without uncertainty measurements	Internal	Variance, variation ratio, and predictive entropy of a distribution of Bayesian probabilities	Good
Hu (30)	Diagnose a rare lymphoma	Positron emission tomography and computed tomography scan images	83 patients	Convolutional neural networks, coarse-to-fine segmentation	Sensitivity 74.7%	Internal	Zone-based uncertainty estimates based on Monte Carlo dropout technique comparing the lesion and the background	Good
Ktena (22)	Evaluate similarity between functional brain networks	Brain functional MRI images	871 subjects	Convolutional neural networks	Overall classification improvement with proposed metric 11.9% and AUC 0.58	External	Calculate similarity between irregular graphs rather than calculating uncertainty directly	Good
Lee (43)	Quantify uncertainty in brain metabolite identification	MRI, proton magnetic resonance spectroscopy	15 rats	Convolutional neural networks	Measurement uncertainty for five major metabolites was less than 10%	Internal	Calculate Cramer-Rao-lower-bounds statistics to estimate the reliability of fitting	Fair
Leibig (44)	Diagnose diabetic retinopathy	Fundus images	89,902 images	Convolutional neural networks	>85% sensitivity and 80% sensitivity when referring 20% of the most uncertain decisions for further inspection	External	Draw Monte Carlo samples from the approximate predictive posterior, use its standard deviation to represent uncertainty	Good
McKinley (45)	Detect multiple sclerosis lesion changes	MRI images	Training: 4–5 sets of 176 images for 26 patients, testing: 77 image sets	Convolutional neural networks	Accuracies of 75% and 85% in separating stable and progressive time-points	External	Use best-practice standards to annotate lesions, predict the probability that a convolutional neural network will assign a different label than assigned a ground truth	Good
Nair (36)	Detect multiple sclerosis lesions	MRIs from patients with relapsing-remitting multiple sclerosis	1064 patients, annual MRIs during a 24-month period	Convolutional neural network	Overall lesion-level true positive rate of 0.8 at 0.2 false detection rate	External	Approximate probability distributions with Monte Carlo dropout and measure their variance, predictive entropy, and mutual information	Good
Natekar (37)	Classify brain tumors	Brain MRI images	Training: 285 cases, testing: 48 volumes	Convolutional neural networks	Whole tumor Dice coefficient 0.830	External	The mean of the variance in a predicted posterior distribution generated by running a model for 100 epochs for each image	Fair
Qin (16)	Estimate brain and cerebrospinal fluid intracellular volume	Brain diffusion MRI scans	Approximately 1,000,000 images (not specified fully)	Convolutional neural network	All correlations between estimation uncertainty and error were significant (*p*<0.001)	External	Train an ensemble of deep networks, measure variance in their fused results	Good
Roy (46)	Identify brain structures	Brain MRIs	Four datasets with MRIs from 30, 29, 13, and 18 subjects	Convolutional neural network	Dice = 0.88, 0.83, 0.81, 0.81	External	Samples are passed through the neural network serially, some weights dropped each time, derive voxel-wise and structure-wise uncertainty from variance across runs	Good
Sedghi (23)	Model agreement for brain image classifications	Brain MRIs	115 subjects	Convolutional neural network	Intra-subject dice for gray matter, white matter, cereprospinal fluid = 0.70, 0.77, 0.62	External	Calculate variance in displacements for different image classifications	Good
Seebock (38)	Detect anomalies in retinal optical coherence tomography images	Optical coherence tomography B-scans	226, 33, 31	Bayesian U-Net, convolutional neural network-based	Precision = 0.748, recall = 0.844, Dice = 0.789	External	Testing samples are passed through the neural network several times, some weights are dropped each time, uncertainty is derived from variance across runs	Good
Tanno (17)	Differentiate among healthy brain, glioma, and multiple sclerosis	Diffusion tensor images or mean apparent propagator-MRI	Training: 16 subjects, validation: variable, overall 28 subjects	Convolutional neural network	Uncertainty-based classification correctly identified 96% of all high-risk (uncertain) predictions	External	Integrate intrinsic uncertainty with a heteroscedastic noise model and parameter uncertainty with Bayesian inference	Good
Valiuddin (18)	Density modeling of medical images	Thoracic computed tomography and endoscopic polyp images	1,108 thoracic computed tomography scans, 1,000 polyp images	Probabilistic U-Net	Increased predictive performance (GED and IoU) of up to 14% with an approach that models uncertainty	External	Learn aleatoric uncertainty as a distribution of possible annotations using a probabilistic segmentation model	
Wang (33)	Classify diabetic macular edema	Optical cohere tomography images	5,028 images	Convolutional and recurrent neural networks	Accuracy 0.951, F1-score 0.935–0.939, AUC 0.986–0.990	External	Mean and standard deviation of probabilistic predictions yielded by ensemble of models	Good
Wickstrøm (47)	Classify polyps seen on colonoscopy	Images obtained from colonoscopies	912 images	Fully convolutional network	IoU background = 0.946, IoU polyp = 0.587, mean IoU = 0.767, global accuracy = 0.949	Internal	Monte Carlo dropout to approximate Bayesian posterior of weights, Monte Carlo-guided backpropagation, standard deviation of pixels	Good
Wieslander (19)	Investigate drug distribution on lung microscopy images	Rat lungs after treatment with different doses and routes of a medication	1,105 images	Convolutional neural network	Precision = 0.89, recall = 0.87, F1 = 0.87; conformal prediction R^2^ = 0.99 for actual vs. observed error	Internal	Conformal prediction using largest p-value minus second largest p-value	Good
*Non-imaging applications*								
Cortes-Ciriano (24)	Drug discovery	Potency of a substance in inhibiting a biochemical or biological function	24 protein drug targets, 203–5,207 bioactivity data points per protein	Ensembles of 100 deep neural networks	Strong correlation between confidence levels and percentage of confidence intervals encompassing true bioactivity (R^2^ > 0.99, *p*<0.001)	External	Ensemble deep neural networks by recording network parameters throughout local minima during single network optimization, calculate variability and validation residuals across snapshots	Good
Cortes-Ciriano (27)	Drug discovery	Potency of a substance in inhibiting a biochemical or biological function	24 protein drug targets, 479–5,207 bioactivity data points per protein	Deep neural networks and random forest	Strong correlation between confidence levels and error rates (R^2^ > 0.99, *p*<0.001)	External	Conformal prediction to compute prediction errors on ensembles of predictions generated by dropout	Good
Scalia (25)	Predict molecular properties	Molecular graphs	4 datasets: 130828, 103657, 11908, and 4200 graphs	Graph convolutional neural networks	Test set errors for 4 datasets: 0.74, 0.32, 1.33, 0.481	External	Monte Carlo dropout, deep ensembles, and bootstrapping with comparison of these three methods	Good
Sieradzki (48)	Compound bioactivity prediction	Bit strings representing compound structures	Several sample sizes, largest: approximately 4,000	Multi-layer perceptron	Models incorporating uncertainty information gained 0.004–0.007 precision	External	Pass test samples through the neural network serially, some weights dropped each time, uncertainty derived from variance in dropout	Good
Teng (28)	Predict Alzheimer’s and Parkinson’s disease progression	Clinical, imaging, genetic, and biochemical markers of neurodegenerative disease	Alzheimer’s: 1,574 patients, Parkinson’s: 1,093 patients	Deep generative model with recurrent neural networks	Alzheimer’s: accuracy = 0.916, AUC = 0.981, F1 = 0.916; Parkinson’s: accuracy = 0.797, AUC = 0.939, F1 = 0.797	Internal	Ensemble of possible patient forecasts using a generative network	Good
Zhang (26)	Predict toxicity for chemical compounds	Toxicities of chemical compounds on nuclear receptors and stress response-related targets	Active class: 7039; inactive class: 89,922	deep neural networks, random forest, light gradient boosting machine	Average AUC = 0.734; single-label predictions generated for about 90% of all instances with overall confidence 80% or greater	External	Conformal prediction using user-defined significance levels	Good

AUC: area under the receiver operating characteristic curve, GED: generalized energy distance, IoU: intersection over union, MRI: magnetic resonance imaging.

## Data Availability

All data are in the manuscript and/or supporting information files.
