# Supplementary material for: Uncertainty-aware deep learning in healthcare: A scoping review
Source: PLOS Digit Health. 2022 Aug 10;1(8):e0000085. doi: 10.1371/journal.pdig.0000085 (PMC9802673; doi:10.1371/journal.pdig.0000085)
Supplement: S1 PRISMA Checklist — (DOCX) [file pdig.0000085.s001.docx]

**S1 PRISMA Checklist.** Preferred Reporting Items for Systematic Reviews and Meta-Analyses extension for Scoping Reviews (PRISMA-ScR) checklist.

| **Section** | **Item** | **PRISMA-ScR Checklist Item** | **Description of Compliance with Checklist Items** |
| --- | --- | --- | --- |
| **Title** | 1 | Identify the report as a scoping review. | The subtitle identifies the report as a scoping review |
| **Abstract** |  |  |  |
| Structured  summary | 2 | Provide a structured summary that includes (as applicable) background, objectives, eligibility criteria, sources of evidence, charting methods, results, and conclusions that relate to the review questions and objectives | A structured summary is included. |
| **Introduction** |  |  |  |
| Rationale | 3 | Describe the rationale for the review in the context of what is already known. Explain why the review questions/objectives lend themselves to a scoping review approach | The Introduction section provides a rationale. |
| Objectives | 4 | Provide an explicit statement of the questions and objectives being addressed with reference to their key elements (e.g., population or participants, concepts, and context) or other relevant key elements used to conceptualize the review questions and/or objectives. | Objectives are listed in Supplemental Figure 1. |
| **Methods** |  |  |  |
| Protocol and  registration | 5 | Indicate whether a review protocol exits; state if and where it can be accessed (e.g. a Web address); and if available, provide registration information, including the registration number. | The Methods refers to a review protocol illustrated in Supplemental Figure 1. |
| Eligibility criteria | 6 | Specify characteristics of the sources of evidence used as eligibility criteria (e.g., years considered, language, and publication status), and provide a rationale. | Eligibility criteria and rationales are provided in Supplementary Figure 1. |
| Information sources | 7 | Describe all information sources in the search (e.g., databases with dates of coverage and contact with authors to identify additional sources), as well as the date the most recent search was executed. | Information sources and the date of the most recent search are provided in the Methods section and in Supplementary Figure 1. |
| Search | 8 | Present the full electronic search strategy for at least 1 database, including any limits used, such that it could be repeated. | Search criteria for three databases are provided in the Methods section and in Supplementary Figure 1. |
| Selection of sources of  evidence | 9 | State the process for selecting sources of evidence (i.e. screening and eligibility) included in the scoping review. | The process for selecting sources is described in the Methods section and in Supplementary Figure 1. |
| Data charting process | 10 | Describe the methods of charting data from the included source of evidence (e.g., calibrated forms or forms that have been tested by the team before their use, and whether data charting was done independently or in duplicate) and any processes for obtaining and confirming data from investigators. | Methods for charting data are described in the Methods section. |
| Data items | 11 | List and define all variables for which data were sought and any assumptions and simplifications made. | Data items are described in the Methods section and in Supplementary Figure 1. |
| Critical appraisal of  individual sources of  evidence | 12 | If done, provide a rationale for conducting a critical appraisal of included sources of evidence; describe the methods used and how this information was used in any data synthesis (if appropriate). | A critical appraisal of individual sources is described in the Methods section. |
| Summary measures | 13 | Not applicable for scoping reviews. | Not applicable for scoping reviews. |
| Synthesis of  results | 14 | Describe the methods of handling and summarizing the data that were charted. | Methods for handling and summarizing data are described in the Methods section. |
| Risk of bias across  studies | 15 | Not applicable for scoping reviews. | Not applicable for scoping reviews. |
| Additional analyses | 16 | Not applicable for scoping reviews. | Not applicable for scoping reviews. |
| **Results** |  |  |  |
| Selection of sources of  evidence | 17 | Give numbers of sources of evidence screened, assessed for eligibility, and included in the review, with reasons for exclusions at each stage, ideally using a flow diagram. | This information is included in a flow diagram (Supplementary Figure 1). |
| Characteristics of  sources of evidence | 18 | For each source of evidence, present characteristics for which data were charted and provide the citations. | Data from all sources of evidence are listed in Table 1. |
| Critical appraisal within  sources of evidence | 19 | If done, present data on critical appraisal of included sources of evidence (see item 12). | A critical appraisal of individual sources is described in the Methods section. |
| Results of individual  sources of evidence | 20 | For each included source of evidence, present the relevant data that were charted that relate to the review questions and objectives. | Data from all sources of evidence are listed in Table 1. |
| Synthesis of results | 21 | Summarize and/or present the charting results as they relate to the review questions and objectives. | Data from all sources of evidence are listed in Table 1 and synthesized into categories in the Results section. |
| Risk of bias across  studies | 22 | Not applicable for scoping reviews. | Not applicable for scoping reviews. |
| Additional analyses | 23 | Not applicable for scoping reviews. | Not applicable for scoping reviews. |
| **Discussion** |  |  |  |
| Summary of evidence | 24 | Summarize the main results (including an overview of concepts, themes, and types of evidence available), link to the review questions and objectives, and consider the relevance to key groups. | Main results are summarized as they are presented in the Results section. |
| Limitations | 25 | Discuss the limitations of the scoping review process. | Limitations are discussed in the Methods section. |
| Conclusions | 26 | Provide a general interpretation of the results with respect to the review questions and objectives, as well as potential implications and/or next steps. | This information is provided in the Conclusions section. |
| **Funding** | 27 | Describe the sources of funding for the included sources of evidence, as well as sources of funding for the scoping review. Describe the role of the funders of the scoping review. | The included studies did not involve commercial products for which sources of funding conferred any conflicts of interest. Sources of funding for the scoping review are provided in the Acknowledgements section. |
