# Supplementary material for: Uncertainty-aware deep learning in healthcare: A scoping review
Source: PLOS Digit Health. 2022 Aug 10;1(8):e0000085. doi: 10.1371/journal.pdig.0000085 (PMC9802673; doi:10.1371/journal.pdig.0000085)
Supplement: Attachment — Submitted filename: Response to reviewer comments R1.docx [file pdig.0000085.s002.docx]

Reviewers' comments:

Reviewer's Responses to Questions

Is the manuscript technically sound, and do the data support the conclusions? The manuscript must describe methodologically and ethically rigorous research with conclusions that are appropriately drawn based on the data presented


Reviewer #1: Yes

Reviewer #2: Partly

Reviewer #3: Yes

--------------------

Has the statistical analysis been performed appropriately and rigorously?

Reviewer #1: N/A

Reviewer #2: N/A

Reviewer #3: N/A

--------------------

Have the authors made all data underlying the findings in their manuscript fully available (please refer to the Data Availability Statement at the start of the manuscript PDF file)?

Reviewer #1: Yes

Reviewer #2: Yes

Reviewer #3: Yes

--------------------

Is the manuscript presented in an intelligible fashion and written in standard English

Reviewer #1: Yes

Reviewer #2: Yes

Reviewer #3: Yes

--------------------

Review Comments to the Author

Please use the space provided to explain your answers to the questions above. You may also include additional comments for the author, including concerns about dual publication, research ethics, or publication ethics. (Please upload your review as an attachment if it exceeds 20,000 characters)

Reviewer #1: The authors address a highly relevant topic by conducting a scoping review on estimation and use of uncertainty around predictions by deep learning algorithms. In the abstract and introduction, they identify mistrust as a barrier to implementing deep learning in the healthcare setting, which I completely agree with.

Major comments:
1) They set the goal to propose a conceptual framework to overcome this barrier, however this section in the Results rather reads like a summary of definitions and methods. I find the introduction of these highly relevant, but I think the concepts should be explained even more thoroughly (with examples), because I imagine that many in the target audience are not aware of these methods. With regard to this, I don't find Figure 1 very intuitive.

**Thank you for making this point. We agree that the results are a bit non sequitur for an introduction focusing mistrust as a barrier to implementing deep learning in the healthcare setting. In writing this manuscript, one of the challenges in bridging the gap between deep learning trust and uncertainty estimations was that uncertainty estimations have not been widely implemented as a means of gaining clinician trust. To address this challenge, we have expanded the first paragraph of the Results section to state that the use of uncertainty estimation in the included articles was rarely applied to building trust in deep learning among patients, caregivers, and clinicians, and that the presentation of results will focus primarily on the content of the articles, and that opportunities to use uncertainty-aware deep learning to build trust is now discussed further in the Discussion section as a novel application of established techniques. As Figure 1 follows the new statement about challenges in bridging the gap between deep learning trust and uncertainty estimations, we hope that the context for Figure 1 will now be stronger, but are happy to omit it if deemed necessary by the Reviewer.** **The Discussion now states that in performing this review we sought to summarize evidence regarding the efficacy of uncertainty estimation in building trust in deep learning among patients, caregivers, and clinicians, but we found little evidence thereof. Therefore, we propose uncertainty-aware deep learning as a novel approach to building trust.**

2) The explanation of epistemic and aleatoric uncertainty is somewhat confusing. In the abstract, the authors write "Overall, while uncertainty estimation accurately quantified aleatoric uncertainty", based on the Results section it seems like "Aleatoric uncertainty is difficult to address directly". Also, they authors claim that epistemic uncertainty can be reduced "by adding more training data", then they write that aleatoric uncertainty can be minimized by "broader data collection". It is not necessarily wrong logic, could be true for both, it just reads a bit confusing in contrasting the two uncertainties and highlighting the same solution.

**Thank you for the opportunity to clarify. We have revised this section to offer the perspective that epistemic and aleatoric uncertainty have overlapping etiologies, as variability and noise in data can contribute to uncertainty regarding optimal model parameters and knowledge regarding ground truth. In addition, epistemic and aleatoric uncertainty may be amenable to similar mitigation strategies, as collecting and analyzing more data may allow for more effective identification and imputation of outlier and missing values, thus reducing aleatoric uncertainty, and may also allow for more effective parameter searches. We then state that beyond these overlapping etiologies and mitigation strategies, epistemic and aleatoric uncertainty have some unique and potentially important attributes, and discuss those attributes. To address some of the confusion on these topics in the original manuscript, we have omitted the statement about how aleatoric uncertainty is difficult to address directly as well as the statement about uncertainty estimations accurately quantifying aleatoric uncertainty.**

3) I completely agree with the authors stating that comparing results is difficult due to different performance metrics across studies, and therefore I would tone down this part in the Results section and suggest to move sentences like "tended to have smaller sample sizes" etc. to the Discussion.

**We have omitted the comparison of predictive performance by sample size in the Results section and moved those references to the Discussion after noting that unsurprisingly, for medical imaging applications, larger datasets of training images were associated with greater predictive performance.**

4) The methods are introduced to tackle mistrust, improvement of predictive performance is really highlighted in the Introduction, however in the Results, this aspect gets quite a bit of attention. Is there a difference between strong predictive performance of a model and strong performance in estimating uncertainty? It would be relevant to explain it to readers. Is improved performance e.g. with the MC method due to an ensemble effect? Some readers new to deep learning might understand this better with an example, analogy (decision tree - random forest, if this fits the purpose).

**Thank you for raising this important perspective. As above, the revised manuscript clarifies that in performing this review we sought to summarize evidence regarding the efficacy of uncertainty estimation in building trust in deep learning among patients, caregivers, and clinicians, but we found little evidence thereof. Therefore, we propose uncertainty-aware deep learning as a novel approach to building trust, and have modified the Results and Discussion section accordingly. Regarding the difference between strong predictive performance of a model and strong performance in estimating uncertainty, we have expanded the description of the study by Valiuddi et al, in which Monte Carlo dropout methods generated accurate uncertainty estimations and also were associated with increased overall model predictive performance, by stating that these findings corroborate prior evidence that Monte Carlo dropout can also offer predictive performance advantages, especially on external validation, by mitigating risk for overfitting. In addition, we have modified the second sentence of the Discussion to highlight that uncertainty estimation methods can simultaneously improve predictive performance (presumably by mitigating risk for overfitting, in the case of Monte Carlo Dropout) and accurately estimate uncertainty. Overall, the manuscript text has been expanded and lengthened in the revision process so we hesitate to add another analogy but would be happy to do so if deemed necessary by the Reviewer.**

5) In the title, the authors define the healthcare domain, therefore I suggest the exclusion of articles on oil prices, fruit images etc. Steinbrener (25), Akbari (39), Wang (41). They could be mentioned in the Discussion, but not as included articles in the scoping review.

**Given the topic it does seem appropriate to move these articles to the Discussion, and we have done so. Table 1 has also been updated to omit these three studies.**

Minor comments:
6) I suggest the use of the PRISMA-ScR Flow Chart in its original format and the inclusion of this in the main body of the article.

**We have used the PRISMA-ScR Flow Chart in its original format and included it in the main body of the article.**

7) Reporting of frequencies and percentages could be slightly improved if written out more precisely, e.g. 11 out of 12 (92%) in the first sentence in Medical Imaging section that refers to another number (12) in that sentence as the 100% and not all 25 articles.

**We have reported the frequencies and percentages by writing out the raw numbers in the first sentence, as described by the Reviewer**.

**Thank you to the Reviewer for your thoughtful comments and critiques. We believe that in addressing them, the manuscript has been improved substantially.**

Reviewer #2: This study aimed to literature review of uncertainty-aware deep learning techniques for healthcare application. I have the following suggestions.
1.      What is the novelty of this study although several literature reviews of Uncertainty-aware deep learning techniques for healthcare data have been done earlier?

**Thank you for pointing out the importance of novelty in this study. As noted in the Discussion, we found no previous systematic or scoping reviews on the same topic, though several authors have described important components of estimating uncertainty in deep learning predictions. If the Reviewer can point us toward a prior systematic or scoping review on the same topic then we would be happy to address this in the manuscript. We do believe that the use of uncertainty estimations to build trust among patients and clinicians is novel in the sense that empirical investigations on this subject have not yet been reported, and have modified the abstract, main text, and conclusions accordingly.**

2.      The abstract should be rewritten and improved by combining the objectives, short methodology, main review findings, and prospective application.

**The revised abstract is presented without subject headings for objectives, methods, results, and conclusions, in keeping with PLoS Digital Health formatting instructions.**

3.      Introduction section need to be improved. Recent studies related to State-of-art Uncertainty-aware  ML/DL applications utilized in healthcare, need to extensively discussed.

**Thank you for this comment. As a scoping review our intention is to extensively discuss studies identified by the search criteria in the Results section rather than in the Introduction section, but we would be happy to modify the formatting and provide extensive discussion of included studies in the Introduction section if allowed and preferred by the Editorial Office.**

4.      Authors should add the several conceptual diagrams or figures to demonstrate the big picture of the scope of deep learning techniques in healthcare domain.

**Thank you for this comment. Although there have been concerns raised in the review process that the conceptual diagram may not intuitively match the content of the Review, we are requesting the opportunity to retain the conceptual diagram now that the context provided for it has been modified.**

5.      Authors should add a comparative summary the performance measures of the reviewed literatures.

**While we share the perspective that comparative summaries of performance metrics can be useful, we also wish to address a thoughtful request from another Reviewer that we tone down the comparisons between results given the different performance metrics used across different studies, and have moved those elements to the Discussion section after noting that unsurprisingly, for medical imaging applications, larger datasets of training images were associated with greater predictive performance and utility of uncertainty estimations.**

6.      Discussion section need to be extended and improved. Authors should discuss the strength and contradictories of reviewed findings in the discussion section.

**The Discussion section has been extended with the intention of improving it by discussing the potential for Monte Carlo Dropout methods to simultaneously improve predictive performance presumably by mitigating risk for overfitting, in the case of Monte Carlo Dropout) while accurately estimating uncertainty and noting that** **in performing this review we sought to summarize evidence regarding the efficacy of uncertainty estimation in building trust in deep learning among patients, caregivers, and clinicians, but we found little evidence thereof. Therefore, we propose uncertainty-aware deep learning as a novel approach to building trust. With other revisions made to the manuscript, we no longer find substantial contradiction between studies reviewed.**

**Thank you to the Reviewer for your attention to this work.**

Reviewer #3: This article addresses the issue of uncertainty quantification for deep learning predictive models.
These models are known to be very good in terms of predictive performance but also to lack explicability.
Therefore, in a delicate and critical domain such as healthcare, it is more than desirable to quantify the uncertainty associated to predictions even if the model error rate is very low.
For these reasons this manuscript is very interesting and it shows clearly various works in healthcare that are addressing the issue of uncertainty aware deep learning models.
The article is easy to read and to understand.
The subject of the article is in the scope of the journal,
There is no statistical analysis in the article, the authors explain clearly how they include articles in the review, and the table exposed  on the articles give responses to the principle questions we can ask about the articles included on the scoping review.

Best regards,

Rayane ELIMAM

**Thank you for your concise summary and encouraging words.**
